# The Influence of Materials on Footwear Behaviour: A Finite Element Simulation Study

**DOI:** 10.3390/ma16227203

**Published:** 2023-11-17

**Authors:** Arina Seul, Aura Mihai, Mariana Costea, Alexandra Bodoga, Antonela Curteza

**Affiliations:** Faculty of Industrial Design and Business Management, “Gheorghe Asachi” Technical University of Iasi, 700050 Iasi, Romania; arina.seul@academic.tuiasi.ro (A.S.); mariana.costea@academic.tuiasi.ro (M.C.); alexandra.bodoga@academic.tuiasi.ro (A.B.); antonela.curteza@academic.tuiasi.ro (A.C.)

**Keywords:** finite element analysis, simulation, contact interaction, gait biomechanics, footwear

## Abstract

The objective of this study was to analyse the influence of materials and their position within the upper assembly on the behaviour of casual footwear using finite element simulation tools. The study was carried out on three models of casual footwear, which are identical in terms of design lines, varying only in the materials of the upper assembly, namely calfskin leather (M1), knitted fabric (M2), and combination of knitted fabric and calfskin leather (M3). The footwear models were designed according to the design constraints specific to casual footwear. The foot was reconstructed based on the shoe last obtained based on anthropometric data. Material definition, 3D models editing, setting up analysis conditions, and constraints were performed using the Ansys 17.2 software. Gait biomechanics were taken into account to define the loading model, force distribution, force values, and constraints. The study evaluates footwear behaviour in terms of directional deformation (Z axis), equivalent von Mises stress, and equivalent elastic strain distribution. This paper explores a methodology that has the potential to enhance the footwear design and manufacturing process, providing designers with information about the deformations and stress distribution on upper parts of the footwear product.

## 1. Introduction

Footwear performance refers to the overall functionality and effectiveness of the product in serving its intended purposes. It encompasses various aspects related to how well the footwear performs in providing protection, comfort, support, and functionality to the wearer. These critical functions rely on the properties of two fundamental subassemblies: the upper and the bottom of the footwear. Evaluating and testing footwear performance is essential to ensure these functions are properly executed and to analyse product design.

While extensive examples of research have examined bottom assembly performance in relation to the foot, including factors like hardness [1], thickness [2], material properties [3], and sole/midsole design [4], and their influence on general comfort perception, the connection between the uppers and the foot remains a challenging area due to experimental control difficulties [5,6,7]. Nevertheless, the significance of uppers on footwear performance, as well as on users’ kinematic and kinetic strategies, cannot be disregarded, given the large contact area between the upper and the foot, along with variations in materials and design lines that influence how the footwear is perceived [5].

Footwear evaluation can be conducted through experimental tests and/or virtual simulations. The latter has gained immense popularity across various fields, including the footwear industry. In contrast to time-consuming and expensive experimental tests, virtual simulations offer cost-effectiveness, rapid scenario analysis, and extensive pre- and post-processing capabilities for optimizing both design and product quality in the product development stage [8,9].

Finite Element Analysis (FEA) serves as a common simulation and analysis method, allowing the replication of structures with irregular geometry, complex material properties, and simulations of loading conditions with variable parameters.

In the footwear field, FEA provides detailed insights into phenomena like internal loading conditions of the human foot [10], plantar pressure distribution [11], stress distributions [12], deformations for different heel heights [13], the impact of bottom assembly materials on plantar pressure distribution and assembly deformation [14,15,16], effects of sole designs on traction performance [9], shock absorption, bending, torsional characteristics [17], impact resistance of intermediate components [18], and heat exchange [19].

These findings highlight that there are limited studies evaluating changes in the upper within entire footwear simulations [20]. However, it is crucial to acknowledge that the materials and design specifics of the upper significantly influence footwear performance and comfort characteristics.

For instance, FEA has been used to simulate the deformation of materials in shoe uppers and evaluate force distribution on the foot surface during a complete step, comparing the influence of three materials on foot and footwear stress distribution. The upper was treated as a uniform surface with minimal thickness, experiencing bending and plane stress in a three-dimensional space. Small strains and, consequently, attributed linear elastic properties to the upper were assumed. This required specifying only three parameters: thickness, Young’s modulus, and Poisson’s ratio. The paper points out that the materials are specific to the footwear industry, without mention of their type [21].

Another study presents the deformation of calfskin, a common material used in shoe uppers, during the gait cycle. The material behaviour proposed for the specified operating conditions was an orthotropic linear elastic model, capable of accommodating large deformations and subjected to membrane and bending loads [22].

Further research introduces the 3D FE coupled model of the foot and sports shoe complex during balanced standing [23]. The material properties were defined using two constants, namely Young’s modulus (E) and Poisson’s ratio (ν), to describe their behaviour. The entire upper assembly was considered as a single component with homogeneous properties over its entire surface.

The literature review emphasises that the studied research assumes the upper to be composed entirely of leather, overlooking the fact that the upper assembly consists of multiple parts, which may be made from various materials.

Considering the subject within the context of diversity and advancements in materials technology, other material, such as synthetic leather, woven, and knitted fabric, must also be taken into consideration. Knitted fabrics have gained popularity as upper materials in both sports and fashion footwear, with companies like Nike and Adidas launching footwear featuring spatial knitted uppers [24]. These knitted fabrics offer increased wearing comfort and a reduction in waste during production compared to leather footwear.

Notably, knitted fabrics like 3D knitted ‘a-jours’ fabric are suitable for footwear linings, reducing discomfort during physical activity due to their spatial warp structure [25]. Regarding full-knitted uppers, interlock flat-knitted spacer fabrics [26] and sock-like jersey-based fabrics are commonly used [27].

It is worth mentioning that there is a lack of studies simulating the behaviour of footwear with knitted uppers. Research has predominantly focused on knitwear performance in other contexts, such as breast-shaping effects and skin pressure distribution by bras [28,29], interactions between the lower-limb musculoskeletal system and compression garments [30], and the pressure impact of highly elastic knit compression underwear on the body [31].

Footwear with knitted uppers is still a challenge for the footwear industry, both in terms of design and manufacture [27]. Therefore, it is found that simulating the behaviour of knitted footwear in the development phase using FEA tools has significant potential in footwear engineering, enabling performance evaluation and optimisation by predicting product behaviour during development. These simulations offer valuable insights into how material combinations and design decisions influence the final footwear product.

Taking the previously mentioned factors into account, this study’s objective is to investigate the impact of materials, specifically calfskin leather and knitted fabric, as well as their allocation to specific upper parts, on footwear behaviour during walking. This aspect deepens designers’ understanding of footwear performance, specifically regarding material variations and their positioning within the upper assembly.

## 2. Materials and Methods

**Figure 1:**
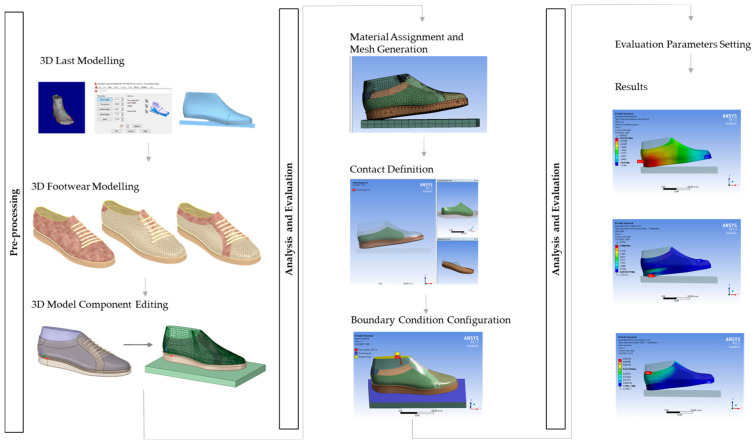
Methodological framework.

This study has been conducted following the methodology represented in Figure 1, which includes pre-processing, analysis, and evaluation stages.

### 2.1. Equipment

To simulate footwear behaviour accurately, it is essential to create appropriate geometric models. This process involves creating three-dimensional (3D) geometric shapes, including the shoe last, the foot, and the various components of the footwear, and then simulating their behaviour using high-performance computer tools. To obtain the 3D shape of the foot, the Infoot USB system (I-Ware Laboratory Co Ltd., Minoh, Japan) was used, which includes a 3D scanner and dedicated software. The mean values of the anthropometric study served as the basis for modelling the appropriate shoe last using Delcam Crispin LastMaker Pro 2015 R2 (Autodesk Inc., San Francisco, CA, USA). Footwear modelling was accomplished using the Delcam Crispin ShoeMaker Pro 2015 R2 software (Autodesk Inc., San Francisco, CA, USA). Finite element analysis was conducted using the Ansys 17.2 Academic Research Mechanical software (ANSYS Inc., Canonsburg, PA, USA).

### 2.2. Three-Dimensional (3D) Model Creation and Editing

The 3D shape of the foot was obtained based on the anthropometric assessment and analysis of the feet of a group of 32 females, aged 18–30 years, with an average body mass of 52 kg falling within the height range of 153–175 cm. None of the subjects had any disorders, and they were not engaged in high-performance sports or other activities that could influence anthropometric foot analysis. Eight anthropometric parameters were measured and analysed, including foot length, ball girth circumference, foot breadth, instep circumference, toe I angle, toe V angle, toe I height, and toe V height. These parameters are of significant importance from a footwear design perspective, aiding in the design or selection of an appropriate last [32]. To characterise and quantify the extent of variation in these values, the main statistical indicators used included the arithmetic mean, standard deviation, sample variance, minimum and maximum values, amplitude of variation, and coefficient of variation. Subsequently, a one-dimensional distribution of anthropometric parameters, the boundaries of frequency classes, the class centre, and the relative and absolute frequency were calculated to describe the average representative foot specific to the analysed group of subjects. The midpoint of the class with the maximum absolute frequency was considered representative for the chosen target group. The mean size resulted from the statistical analysis was 37 in the French system and 240 in the metric system. The values obtained were used to create the last using Delcam Crispin LastMaker Pro 2015 R2 (Autodesk Inc., USA) as it is presented in previous conducted research [33].

To assess how the materials of the upper part influence the behaviour of the footwear during walking, a classic version of casual footwear was chosen, whose upper assembly includes the following parts: vamp, tongue, front facing, quarter, collar, back strip, and back tab facing. This upper structure was selected to enable the customisation and optimisation of the model concerning the materials used for the specific parts of the footwear product.

Footwear modelling was performed using the Delcam Crispin ShoeMaker Pro 2015 R2 software (Autodesk Inc., San Francisco, CA, USA). The 3D footwear design started by importing the last developed in the previous stage. Next, the primary reference and design lines were drawn. From a design point of view, the quarter line was raised by 15 mm in the heel area, compared to the regular shoes. To ensure an easy shoe fit, the opening vamp point has been moved 10 mm towards the toe. Based on these lines, each upper part was created, and the sole was generated.

**Figure 2:**
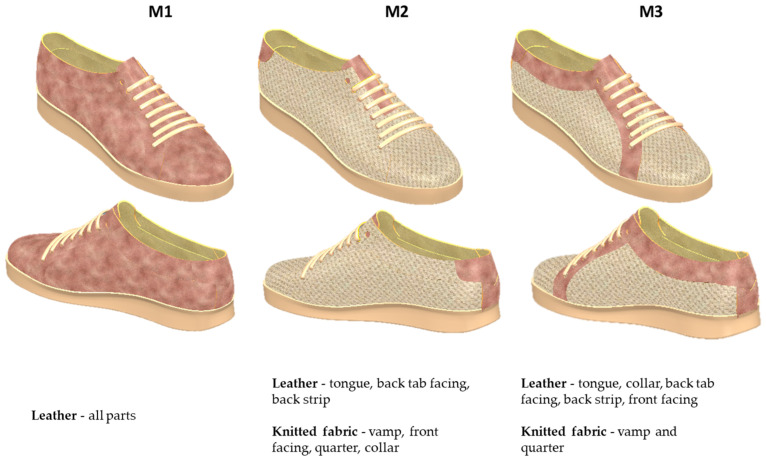
Casual footwear—3D models.

At the end of this stage, three footwear models were obtained. The models are identical in terms of design lines, varying only in the materials used for the upper assembly, as shown in Figure 2.

Two materials were chosen, namely calfskin leather and a single jersey knitted fabric. Both materials provide good properties in terms of comfort, breathability, and moisture management. However, their elasticity, deformability, and resilience are significantly different, which is a challenge in terms of footwear product design and manufacture.

All the geometric components were edited using the tools provided by the Space Claim application, which is integrated into the Ansys 17.2 software. The foot was reconstructed based on the last imported in *.stl format. The parts of the upper assembly were imported in *.iges format and assigned a thickness of 2 mm. The sole was imported in *.iges format and converted into a solid. The entire footwear-foot assembly was placed on parallelepipedal support constructed directly within the Space Claim application.

### 2.3. Properties of Materials

The behaviour of the materials was characterised using two constants: Young’s modulus and Poisson’s ratio [22,23]. The values for Young’s modulus and Poisson’s ratio, specific to the foot, upper assembly, bottom assembly, and support, are provided in Table 1.

In the case of the foot, a basic structure was chosen, omitting consideration of properties related to the muscular and skeleton system, cartilages, and joints [34]. For the sole, a material with properties and characteristics typical of rubber was selected [35].

Concerning the upper assembly components, three scenarios were considered, based on the configurations presented in the 3D modelling stage of the footwear, namely M1 (all upper assembly parts are made of calfskin leather), M2 (tongue, back strip, back tab facing—calfskin leather; vamp, front facing, quarter, collar—knitted fabric), and M3 (tongue, front facing, collar, back strip, back tab facing—calfskin leather; vamp and quarter—knitted fabric).

Footwear represents a structurally complex, multi-layered product, with its constituent parts organised into groups and subgroups. The components of the shoe upper undergo a range of technological processing operations including cutting, stitching, lasting, soling, and finishing. The primary stresses induced by these processes are longitudinal and transversal elongation, stretching and compression, bending, moisture, etc. To withstand these stresses, the materials employed in the upper assembly must demonstrate a specific set of characteristics, which involve elastic-plastic behaviour when subjected to tensile stress, resilience against stretching and tearing, flexibility, and resistance to abrasion. These specifications are met by natural materials, such as leather, as well as by synthetic alternatives to leather or other textile materials and fabrics.

Leather is an anisotropic material which exhibits a broad spectrum of Young’s modulus values, ranging from 20 to 100 N/mm^2^, thus enabling it to manifest elastic deformation characteristics. This property facilitates not only its resilience during the utilisation of footwear but also its ability to partially revert to its initial configuration, a phenomenon observable in both the wearing of the footwear and its production process [22,36,37].

**Figure 3:**
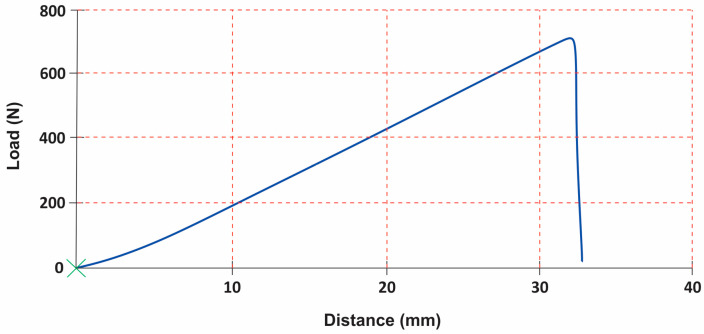
Tensile curve of materials for footwear upper [38].

The tensile curves (Figure 3) of materials used for footwear upper exhibit a nuanced response with characteristics encompassing non-elastic, elastic, and plastic-type behaviours [38]. Consequently, the tensile curve can be divided into distinct regions. The initial phase is marked by a non-elastic response, followed by a subsequent region demonstrating a more stable elastic behaviour.

The knitted fabric used in the simulation is a single jersey, featuring 40D XLA elastic fibres [39]. The entire foot-footwear assembly is placed on a concrete support.

### 2.4. Setting Analysis Conditions

The analysis of the behaviour of the three footwear models was carried out in the Static Structural module of Ansys 17.2 Academic Research Mechanical software (ANSYS Inc., USA). Materials with defined properties in the previous step were assigned to each geometrical component. The thickness of the upper components was set to 2 mm.

Model discretisation was performed using the ‘tetrahedrons’ method commonly used for 3D solid models due to its versatility. The mesh was created automatically on all components using a coarse finite element size and an average alignment of the node positions, resulting in a grid with 131,656 nodes and 77,176 elements. The algorithms of the Ansys 17.2 software adapted the mesh to the geometry and loading conditions, ensuring balance between accuracy and computational efficiency. A direct bonded contact with a tolerance of 0.91 mm was assigned between the different upper parts, as well as between upper and bottom. Frictional contact with a coefficient of 0.6 was applied between the foot and the footwear, as well as between the bottom and the support.

To define the boundary conditions, namely loading pattern, force distribution, and their values, gait biomechanics analysis from previous research was considered [40]. The biomechanical gait analysis involved 32 volunteer subjects, female, 18–30 years old, wearing footwear size numbers 36 (group 1), 37 (group 2), 38 (group 3) in the French system, with an average body mass of 52 kg. None of the subjects had any disorders, and they were not engaged in high-performance sports or other activities that could influence the gait pattern. The study involved the measurement of four fundamental biomechanical parameters: force, pressure, contact time, and contact area. These data were gathered using a pressure platform Footscan 7 Gait Scientific (RS Scan International, Ipswich, United Kingdom) and dedicated software. To ensure consistency, a Student’s *t*-test was applied to confirm the absence of significant differences between the left and right foot. Statistical measures for each parameter were computed, including the mean, standard deviation, minimum and maximum values, amplitude of variation, and coefficient of variation.

**Figure 4:**
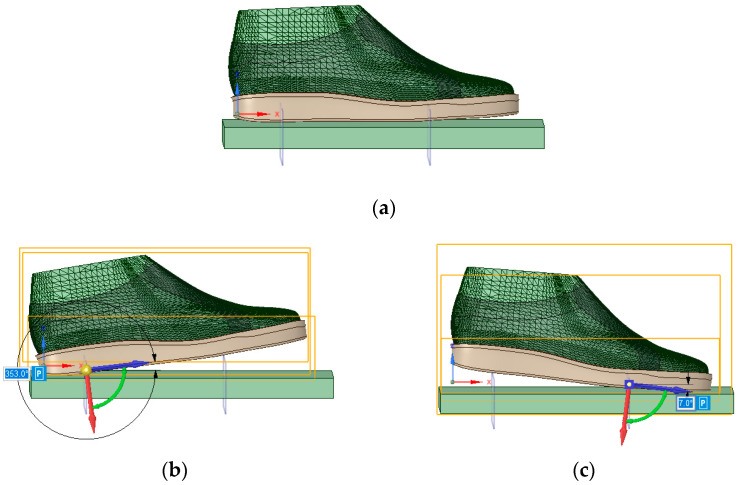
Representation of foot positions in the (**a**) stance, (**b**) heel strike, and (**c**) push-off phases.

The boundary conditions have been established taking into account the three phases of gait, namely heel strike, stance, and push-off. During the stance phase, the foot maintains full contact with the ground plane across its entire plantar surface (Figure 4a). In the heel strike and push-off phases, the foot makes partial contact with the ground plane and is inclined at angles of −7 (Figure 4b) and 7 degrees (Figure 4c) [9]. The pivot point for the heel-strike phase representation was located in the middle of the heel, corresponding to 0.18 of the foot length. For the push-off phase, the pivot point was selected as the centre of the metatarsophalangeal joints, positioned at 0.72 of the foot length.

Human motion comprises a combination of various rotational and translational movements, which result from the interplay of a complex field of forces. These motions result from a combination of internal forces, primarily arising from muscle actions and bone interactions, and external forces, primarily initiating from factors like body weight and ground reaction forces [41]. In the initial heel-strike phase, the weight force acts vertically downward through the centre of mass, with kinetic energy being converted to potential energy as the centre of mass rises. In the subsequent stance phase, the weight force remains vertically downward, offering support for balance and propulsion, particularly during mid-stance when it stabilises the body’s forward movement. Throughout this phase, muscles and joints in the lower extremities counteract the weight force to control and direct movement. In the push-off phase, as the foot leaves the ground and the leg swings forward, the weight force aids in propelling the body. Muscles and tendons in the lower extremities contract to overcome the weight force and initiate forward movement, shifting the weight force to the opposite foot as the gait cycle repeats with each step.

**Figure 5:**
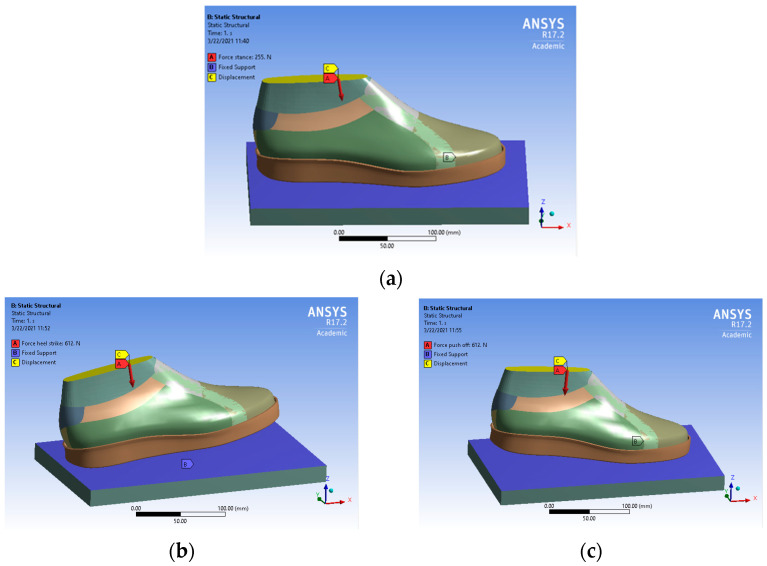
Loading and boundary conditions in the (**a**) stance, (**b**) heel strike, and (**c**) push-off phases.

Based on this, the foot was subjected to a remote force vector in the vertically downward direction to represent the weight force [16,42]. In heel strike and push-off, the body exerts a force equal to 1.2 of weight force [43]. Considering that the average body mass specific to the group of subjects obtained from the biomechanical study is 52 kg, a force of 612 N was applied (Figure 5b,c). In the stance phase, the entire body weight is equally distributed between the two feet. Since the analysis is conducted on one foot, a 255 N force was applied (Figure 5a).

The footwear-foot assembly was allowed to move in the Z direction, while the support was considered fixed. The analysis time was limited to one second. Additional forces were not taken into account in this methodology.

### 2.5. Setting the Parameters to Evaluate

The directional deformation on *Z* axis (mm), equivalent von Mises stress (MPa), and equivalent elastic strain (mm/mm) distribution were evaluated to study the behaviour of the footwear product depending on the type of materials used in the upper assembly. The analysis highlights the displacements of the model, the yield of the materials, as well as the distribution of stress during each phase of gait (heel strike, stance, push-off) over one second, influenced by the weight force.

## 3. Results and Discussion

### 3.1. Results

The results for the directional deformation, von Mises stress, and elastic strain centralized and presented in Table 2, Table 3 and Table 4.

The recorded values are accompanied by images containing an array of colours from blue (low values) to red (high values). This colour coding, accompanied by the numerical values obtained, highlights areas of high stress and deformation of the parts, which have a simultaneous effect during gait on both footwear and foot.

**Figure 6:**
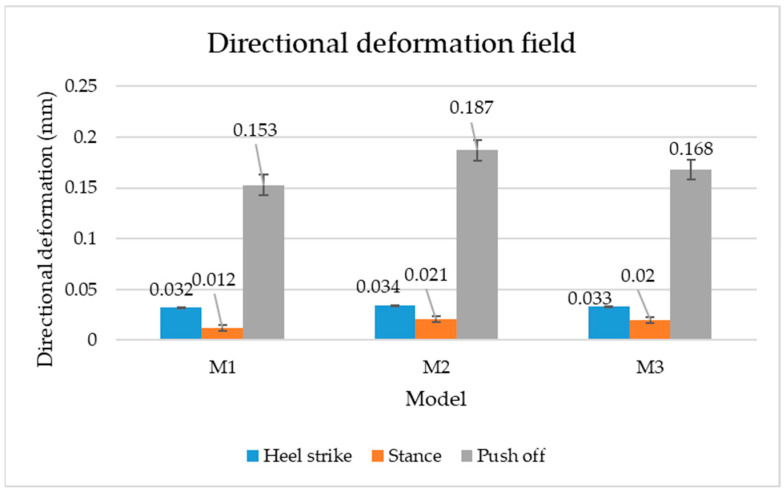
*Z*-axis directional deformation field.

**Figure 7:**
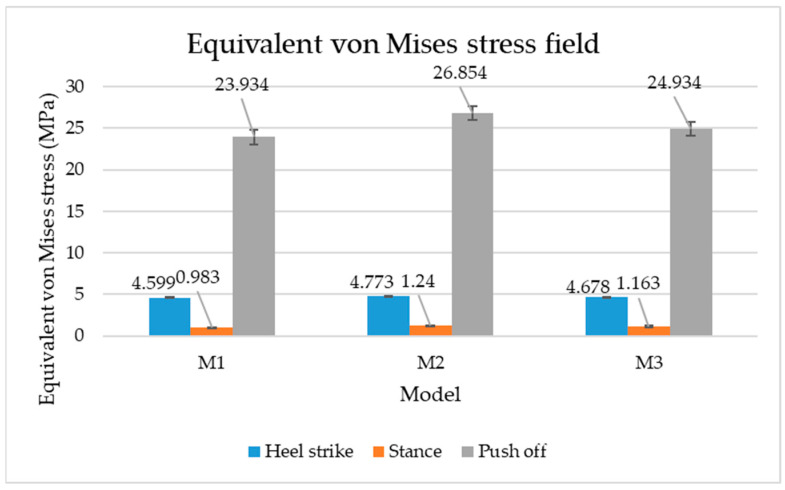
Equivalent von Mises stress field.

**Figure 8:**
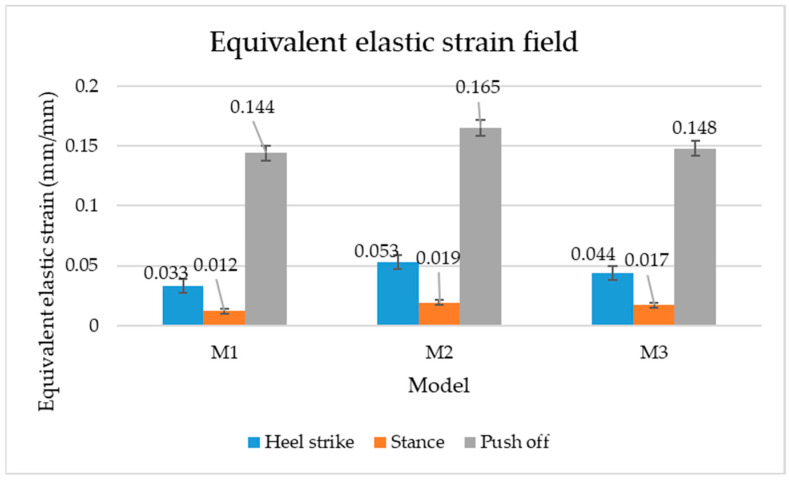
Equivalent elastic strain field.

Figure 6, Figure 7 and Figure 8 show graphically the maximum values of the directional deformation, equivalent von Mises stress, and equivalent elastic strain for the three models in each phase of the gait.

### 3.2. Discussion

The *Z*-axis directional deformation exhibits higher values during the push-off and heel-strike phases when the foot is subjected to more significant forces. This distribution remains consistent across all models under analysis. In the heel-strike phase, M2 exhibits the maximum deformation in the *Z*-axis direction (0.034 mm), followed closely by M3 (0.033 mm) and M1 (0.032 mm). A similar trend is observed in the stance phase, with M2 showing the highest value (0.021 mm), followed by M3 (0.020 mm) and M1 (0.012 mm), respectively.

During the push-off phase, M2 demonstrates the highest maximum value (0.187 mm), followed by M3 (0.168 mm) and M1 (0.153 mm). The chromatic maps indicate that the highest Z-direction deformation values, considering the entire footwear product, occur in the bottom assembly. In the upper assembly, lower directional deformation values are observed, with their distribution varying according to the gait phases, specific areas, and the materials used in those areas.

Comparing the three models during the heel-strike phase, M1, with its upper assembly entirely composed of leather, exhibits lower deformation values. Analysing the chromatic maps reveals that these values increase in the heel area (quarter and back strip) and decrease in the vamp area, front facing, tongue, and collar. In contrast to M1, M2 in the same heel-strike phase displays higher deformation in the forefoot of the footwear, particularly in the vamp and front-facing areas. Simultaneously, it is evident that the minimum deformation occurs at the upper edge of the collar. In the case of model M3, which combines knitted parts (quarter and vamp) with leather parts (tongue, front facing, collar, and back strip), deformations are smaller compared to M2. Notably, there are no significant deformations in the vamp area, and in the collar area, they are reduced even further compared to M1.

During the stance phase, as per the chromatic maps, deformations are smaller and exhibit a more uniform distribution across the footwear’s surface. In the propulsion phase, when forces are concentrated in the toe area, the upper region experiences larger deformations.

An analysis of the values representing the equivalent von Mises stress reveals that the highest pressures are experienced by the footwear product during the push-off phase (23.934, 26.854, 24.934 MPa), followed by the heel-strike phase, which witnesses an approximately 83% reduction (4.599, 4.773, 4.678 MPa), and the stance phase (0.983, 1.240, 1.163 MPa), showing a decrease of 95% compared to the push-off phase. This distribution can be attributed to the substantial deformation of the footwear occurring in the area where the foot bends during the push-off phase, leading to a more uniform distribution during the stance phase—a phenomenon well-documented in the literature [44].

When considering this parameter for each model, the highest values are observed for M2, followed by M3 with an 8% decrease in the push-off phase, 7% in the stance phase, and 2% in the heel-strike phase. For M1, there is an 11% reduction in the push-off phase, 21% in the stance phase, and 1% in the heel-strike phase. According to the chromatic maps, these stresses predominantly manifest in the bottom assembly area. However, it is important to note that all the models under investigation feature soles with identical properties. Consequently, it can be inferred that the material structures within the upper assembly exert a notable influence on the transmission of stresses from the foot to the footwear.

The maximum elastic strain increases during the push-off phase, while during the heel-strike and stance phases, the values of this parameter decrease by approximately 67% and 88%, respectively. Similar to the distribution of values for the equivalent von Mises stress field parameter, higher values for elastic strain are observed in the M2 model (0.053, 0.019, 0.165), followed by the M3 model (0.044, 0.017, 0.148), and the M1 model (0.033, 0.012, 0.144).

The distribution of elastic strain varies by area depending on the gait phase and the type of material. In the heel-strike phase, it is evident that the M2 model exhibits a high elastic strain predominantly in the collar and quarter areas, in contrast to the M1 model, where the values of this parameter are very low across the entire surface of the footwear. The M3 model, on the other hand, shows lower elastic strain in the collar area compared to the same part of the M1 model. In the push-off phase, higher elastic strain is observed in the front part of the footwear, with the maximum strain identified in the area of the front facing. During the stance phase, the elastic deformation values are lower compared to the impact phase and the propulsion phase. Its distribution on the surface of the footwear is mainly concentrated in the central-posterior part (collar, back strip, quarter), with higher values noted for the M2 model.

A comparative analysis of the results obtained with examples of research in the field is limited due to the scarcity of studies on the simulation and evaluation of upper assembly behaviour. In accordance with research conducted by Ruperez et al. [21], stresses occurring in the upper assembly range between 0.9 and 33 MPa, depending on the type of material. According to our results, the stress field displays a similar variation, ranging from 0.983 to 26.854 MPa, contingent upon the gait phase and the types of materials utilised in the upper assembly structure.

Similar results were also presented in another study [44], where the Boundary Element method was applied to compare the behaviour of sports footwear with a court shoe. The maximum pressure values were observed in the push-off phase and amounted to 25.850 MPa for the sports shoe, which closely aligns with the results obtained in the analysis of the present case study (23.934, 26.854, 24.934 MPa).

### 3.3. Validation of the Model Based on Biomechanical Analysis

In a comparative analysis of the impact of footwear on the distribution of dorsal and plantar pressures, existing literature reveals that variations in plantar pressures manifest with greater discernibility than alterations in dorsal pressure distributions across different footwear models [45]. Considering this, the validation of the loading model was performed by comparing the simulated and experimental plantar pressures obtained from the biomechanical study based on walking trials conducted in the previous research [40].

**Figure 9:**
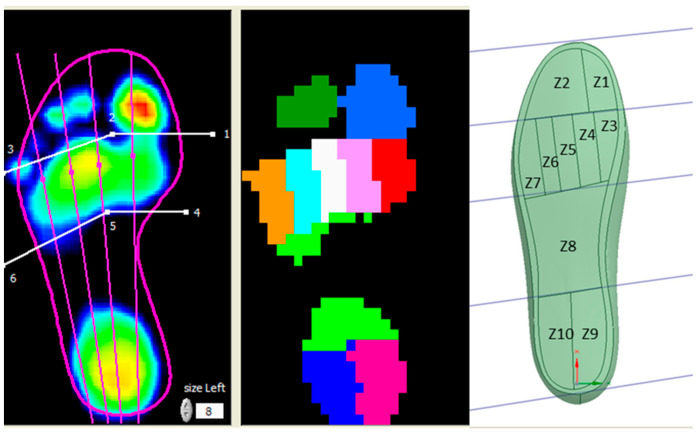
Plantar footprint in relation to foot contour.

To facilitate value comparison and validate the loading model, it was necessary to divide the 3D foot plantar area into ten distinct zones, similar to the Footscan system (Figure 9). Given that the footprint does not precisely match the foot contour, zone delineation was achieved by reducing the footprint by a few millimetres from the edge of the foot contour.

Based on the results of the biomechanical study [40], it was observed that the plantar pressure values in the Z2 zone were exceedingly low, rendering them inconsequential for model validation. As pressures during the stance phase are distributed across the entire plantar surface, comparing them with experimental values would not provide meaningful insights. Therefore, it was decided to focus the comparison on pressures in Z9 and Z10 during the heel-strike phase and pressures in Z1, Z3–Z7 during the push-off phase.

**Figure 10:**
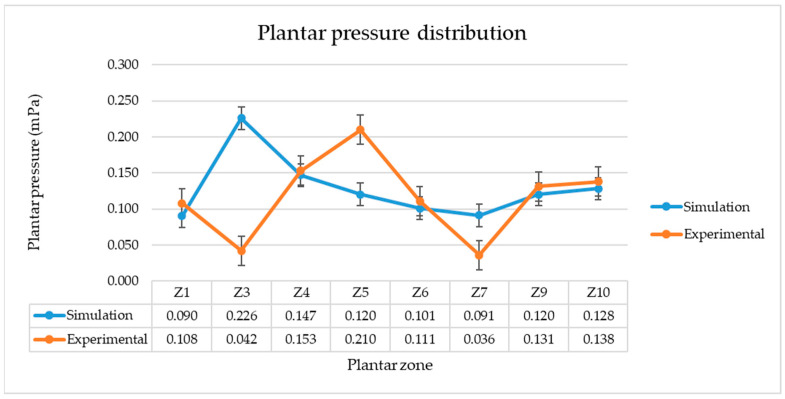
Comparative analysis of plantar pressure distribution values obtained by simulation and experimentally.

The maximum pressure values are presented in Figure 10. It can be seen that for five of the eight zones studied, the simulated maximum pressure values are very close to those obtained experimentally.

## 4. Conclusions

The primary aim of this study was to assess the impact of different materials, as well as their position within the footwear upper, on the performance of casual footwear and its behaviour. Three distinct models of footwear were modelled considering the same design construction but differing in terms of the materials assigned to the upper parts, namely calfskin leather (M1), knitted material (M2), and a combination of knitted material and calfskin leather (M3).

The design of the footwear models adhered to the specifications relevant to casual footwear and was made using Delcam Crispin ShoeMaker Pro 2015 R2 software. The foot was reconstructed based on the shoe last, which was derived from anthropometric data.

Material definition, 3D models editing for compatibility with the simulation application, and establishment of boundary conditions and constraints were performed using Ansys 17.2 Academic Research Mechanical software. The loading model, force distribution, force magnitudes, and constraints were configurated based on gait biomechanics taking into account the forces generated by the bodyweight. The analysis considered three phases of gait: heel strike, stance, and push-off, while evaluating three analysis parameters—directional deformation, equivalent von Mises stress, and equivalent elastic strain distribution. The study provides insights into how these parameters fluctuate depending on the phase of gait and the choice of materials utilised in different parts of upper assembly.

To validate the accuracy of the analysis, a comparison was made between the plantar pressures obtained from the simulation results and the mean plantar pressures obtained from a biomechanical study that assessed the distribution of plantar pressures.

This paper explores a methodology that has the potential to enhance the footwear design and manufacturing process. Compared to other studies, which assessed the upper of the footwear as a homogeneous single-part assembly, this paper takes into account both the structure and the materials assigned to individual upper parts. This aspect enhances designers’ comprehension of footwear behaviour, especially concerning variations in materials and their placement within the upper assembly.

The results highlight that the elasticity of the material has a direct impact on stress distribution and deformation within the shoe. The inclusion of knitted fabric in the footwear structure led to a reduction in stress levels within the overall material assembly. On the other hand, in the case of the model made from knitted fabric (M2), a higher degree of deformation is observed, which can be mitigated by introducing components made from leather (M3).

The study, as well as the methodology presented, has a number of limitations which should be considered in the future work.

Boundary conditions: It is crucial to extend the boundary conditions to include posture and factors related to muscle activity, as these play a significant role in the mechanical behaviour of footwear during various activities.

Mesh refinement: Careful attention should be given to mesh refinement, considering density, quality, and adaptability to specific footwear behaviour. Mesh refinement and validation against experimental data could be used to improve the precision of FEA results in footwear analysis.

Quantifying uncertainty: In future work, quantifying uncertainty in material properties and other input parameters, such as geometry, should be considered to provide a more realistic assessment of the influence of materials [46].

Upper layers: An additional area for potential research involves the detailed examination of the various layers within the upper assembly structure. This encompasses investigating linings, intermediate layers, and the distinctive properties existing within the overlapping zones among the upper’s parts.

## Figures and Tables

**Table 1:** Properties of the materials used in the analysis.

	Foot	Leather (Upper)	Knitted Fabric (Uppers)	Rubber (Bottom)	Concrete (Support)
Young modulus (MPa)	4.47	20	0.0025	1000	90,000
Poisson coefficient	0.45	0.4	0.43	0.42	0.18

**Table 2:** Directional deformation (*Z* axis) values.

Gait Phase	Model M1		Model M2		Model M3	
	Maximum Value (mm)		Maximum Value (mm)		Maximum Value (mm)	
Heel strike	0.032	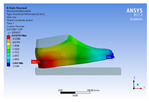	0.034	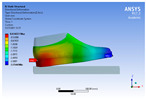	0.033	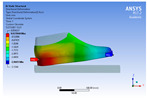
Stance	0.012	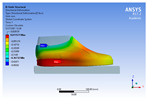	0.021	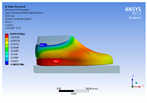	0.020	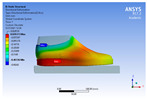
Push-off	0.153	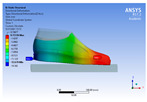	0.187	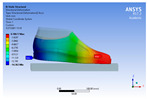	0.168	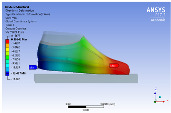

**Table 3:** Equivalent von Mises stress values.

Gait Phase	Model M1		Model M2		Model M3	
	Maximum Value (MPa)		Maximum Value (MPa)		Maximum Value (MPa)	
Heel strike	4.599	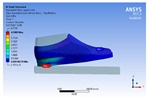	4.773	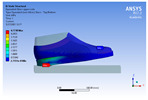	4.678	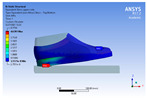
Stance	0.983	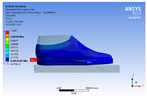	1.240	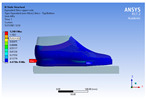	1.163	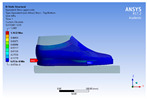
Push-off	23.934	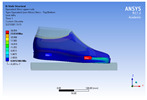	26.854	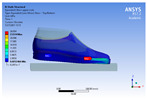	24.934	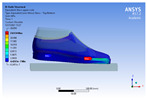

**Table 4:** Equivalent elastic strain values.

Gait Phase	Model M1		Model M2		Model M3	
	Maximum Value (mm/mm)		Maximum Value (mm/mm)		Maximum Value (mm/mm)	
Heel strike	0.033	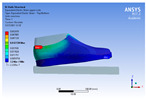	0.053	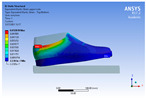	0.044	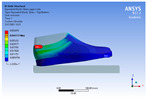
Stance	0.012	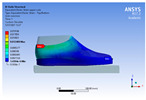	0.019	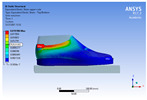	0.017	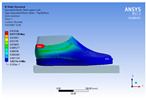
Push-off	0.144	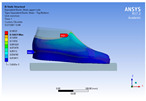	0.165	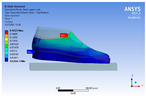	0.148	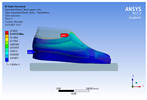

## Data Availability

Data are contained within the article.
